# Pain E-motion Faces Database (PEMF): Pain-related micro-clips for emotion research

**DOI:** 10.3758/s13428-022-01992-4

**Published:** 2022-10-17

**Authors:** Roberto Fernandes-Magalhaes, Alberto Carpio, David Ferrera, Dimitri Van Ryckeghem, Irene Peláez, Paloma Barjola, María Eugenia De Lahoz, María Carmen Martín-Buro, José Antonio Hinojosa, Stefaan Van Damme, Luis Carretié, Francisco Mercado

**Affiliations:** 1 Department of Psychology, School of Health Sciences, Rey Juan Carlos University, Av. Atenas s/n, 28922 Alcorcón, Madrid, Spain; 2 Department of Experimental Health Psychology, School of Psychology and Neuroscience, Maastricht University, Maastricht, Netherlands; 3 Insituto Pluridisciplinar, Universidad Complutense de Madrid, Madrid, Spain; 4 Departamento de Psicología Experimental, Procesos Cognitivos y Logopedia, Universidad Complutense de Madrid, Madrid, Spain; 5 Centro de Investigación Nebrija en Cognición (CINC), Universidad Nebrija, Madrid, Spain; 6 Department of Experimental-Clinical and Health Psychology, Ghent University, Ghent, Belgium; 7 Facultad de Psicología, Universidad Autónoma de Madrid, Madrid, Spain

**Keywords:** Pain-related faces, Emotional expressions, Database

## Abstract

A large number of publications have focused on the study of pain expressions. Despite the growing knowledge, the availability of pain-related face databases is still very scarce compared with other emotional facial expressions. The Pain E-Motion Faces Database (PEMF) is a new open-access database currently consisting of 272 micro-clips of 68 different identities. Each model displays one neutral expression and three pain-related facial expressions: posed, spontaneous-algometer and spontaneous-CO_2_ laser. Normative ratings of pain intensity, valence and arousal were provided by students of three different European universities. Six independent coders carried out a coding process on the facial stimuli based on the Facial Action Coding System (FACS), in which ratings of intensity of pain, valence and arousal were computed for each type of facial expression. Gender and age effects of models across each type of micro-clip were also analysed. Additionally, participants’ ability to discriminate the veracity of pain-related facial expressions (i.e., spontaneous vs posed) was explored. Finally, a series of ANOVAs were carried out to test the presence of other basic emotions and common facial action unit (AU) patterns. The main results revealed that posed facial expressions received higher ratings of pain intensity, more negative valence and higher arousal compared with spontaneous pain-related and neutral faces. No differential effects of model gender were found. Participants were unable to accurately discriminate whether a given pain-related face represented spontaneous or posed pain. PEMF thus constitutes a large open-source and reliable set of dynamic pain expressions useful for designing experimental studies focused on pain processes.

**Supplementary Information:**

The online version contains supplementary material available at 10.3758/s13428-022-01992-4.

## Introduction

One of the main functions of facial expressions is related to the transmission of internal states (Ekman et al., 1972; Redican, 1982; Rosenthal, 2005). According to some views, six basic facial expressions (i.e., happiness, surprise, fear, disgust, anger and sadness) have been proposed as universal emotions across all cultures (Ekman, 1989, 1992a, 1992b, 1994; but see Gendron et al., 2018). Evolutionary-based approaches have argued that this ability to communicate affective and mental states through facial movement configurations is crucial for human adaptation to the environment (Darwin & Prodger, 1998; Fridlund, 2014; Schmidt & Cohn, 2001). Similarly, pain-related faces communicate the state of the pain sufferer through facial movement changes in order to raise alarm in external observers (Prkachin, 1986, 1992; Prkachin & Craig, 1995).

In recent years, a growing number of studies have focused on the processing of facial expressions of pain. The use of this type of stimuli has led to a better understanding of the role of cultural and perceptual factors in the formation of the mental representation of these facial expressions (Chen et al., 2018), and has increased our knowledge regarding the clinical assessment of pain biases (Ashraf et al., 2019; Hirsh et al., 2008; Lucey et al., 2009) or the attentional mechanisms involved in the processing of pain-related faces (Fernandes-Magalhaes et al., 2022; González-Roldán et al., 2013; Heathcote et al., 2015; Khatibi et al., 2009; Vervoort et al., 2013). Importantly, dynamic emotional faces seem to recruit facial processing neural networks more reliably than static facial expressions (Trautmann et al., 2009). Therefore, moving faces seem to intensify the emotional reaction, allow for better recognition of facial expression, and evoke more intense reactions in the viewer, than static faces (Ambadar et al., 2005; Bomfim et al., 2019; Calvo et al., 2016; Trautmann et al., 2009). In the particular case of pain expressions, perceiving the progressive sequence of facial changes enables the observer to more reliably and intensely interpret the internal state and feelings of the pain sufferer (Lucey et al., 2009; Williams, 2002).

The study of the processing of facial expressions of pain significantly relies on the availability of stimuli rated in a number of variables that allow the selection of faces carefully matched on several variables. Although there exist more than 40 facial expression databases (e.g., Ekman, 1976; Georghiades et al., 2001; Goeleven et al., 2008; Lundqvist et al., 1998; see an exhaustive list in www.face-rec.org/databases/), to the best of our knowledge, only four databases include quantitative ratings of pain-related faces (Lucey et al., 2011; Mende-Siedlecki et al., 2020; Simon et al., 2008; Zhang et al., 2014). These databases are a key tool at both the clinical and research levels, and databases including dynamic pain-related facial expressions (Lucey et al., 2011; Simon et al., 2008; Zhang et al., 2014) are of special interest. One includes only pain posed facial expressions (Simon et al., 2008) from eight models—four women and four men (aged 18 to 32 years)—and the other two (Lucey et al., 2011: 129 models—66 women and 63 men; Zhang et al., 2014: 41 models—23 women and 18 men, aged 18 to 29; BP4D-Spontaneous database), only spontaneous expressions. These expressions were elicited by tonic pain stimulation (shoulder injuries) in the Lucey and collaborators (2011) database and phasic pain (cold pressor test) in Zhang and collaborators (2014). In our opinion, the research and clinical practice in this area would benefit from complementing these databases with a comprehensive new database that includes characteristics not covered by them. First, we consider that both spontaneous expressions, due to their ecological value (Craig et al., 1991; Poole & Craig, 1992; Schmidt et al., 2006), and posed expressions, given their controlled nature and ease of identification (Faso et al., 2015; Zhihong Zeng et al., 2009), are valuable and should be included in a new database. Second, both tonic-based (e.g., cold pressor test or algometer) and phasic-based (e.g., CO_2_ laser) pain facial expressions may also be of interest to cover a wider range of expressive variants. Third, extending the age of models in a new database would allow more representative coverage of the general population.

Therefore, the main aim of this study was to provide a new tool, the Pain E-Motion Faces Database (PEMF), containing a large set of pain-related dynamic expressions and their normative ratings. These ratings covered both discrete (i.e., happiness, fear, disgust, surprise, anger and sadness) and dimensional (i.e., intensity, valence and arousal) emotional approaches. The database also offers static facial expressions of pain recorded in the sample of models from youth to old age. Different pain-related faces (spontaneous and posed pain experiences) elicited by tonic and phasic pain stimulation are provided as well. Additionally, we code facial action units from the pain-related faces through the Facial Action Coding System (FACS; Ekman & Friesen, 1977). This tool allows us to characterize facial movements linked to emotional expressions (called facial action units, AUs).

## Methods

### Stimuli

Sixty-eight models (23 men and 45 women), aged 18–61 years (mean = 30.34; SD = 12.26), took part in the session for creating pain-related micro-clips. The sample was distributed among adult women (56%; age range 18–44), adult men (25%; age range 18–44), elderly adult women (10%; age range 45–61) and elderly adult men (9%; age range 45–61). Recruitment was carried out at Rey Juan Carlos University (Madrid, Spain) using a snowball sampling procedure. Before the start of the session, all participants were informed about the whole procedure and signed an informed consent form. The study was approved by the Rey Juan Carlos University Research Ethics Committee. Participants provided their written consent after being informed of the details of the procedure, and were made aware that micro-clips collected could be used for research goals (i.e., journal articles, scientific conferences, meetings, experimental designs), and might be manipulated (i.e., luminosity, size, labelled indicating group membership) for those purposes. They were told that no personally identifiable data other than their emotional expression would be published (i.e., name or place of residence). They could stop the session at any time, including removing informed consent for the use of clips without any ethical or economic prejudice.

The video sequence session was carried out in a room with optimal insulation conditions for acoustic and electromagnetic signals. Participants were seated facing a camcorder (Sony Handycam HDR-XR550VE camera) located at eye level, at a distance of 50 cm, to continuously record the entire facial expression. The filming team consisted of two research assistants. To minimize possible interference of external facial elements, research assistants asked participants to remove any special face cues such as piercings, glasses, earrings or any other distinctive object.

**Fig. 1:**
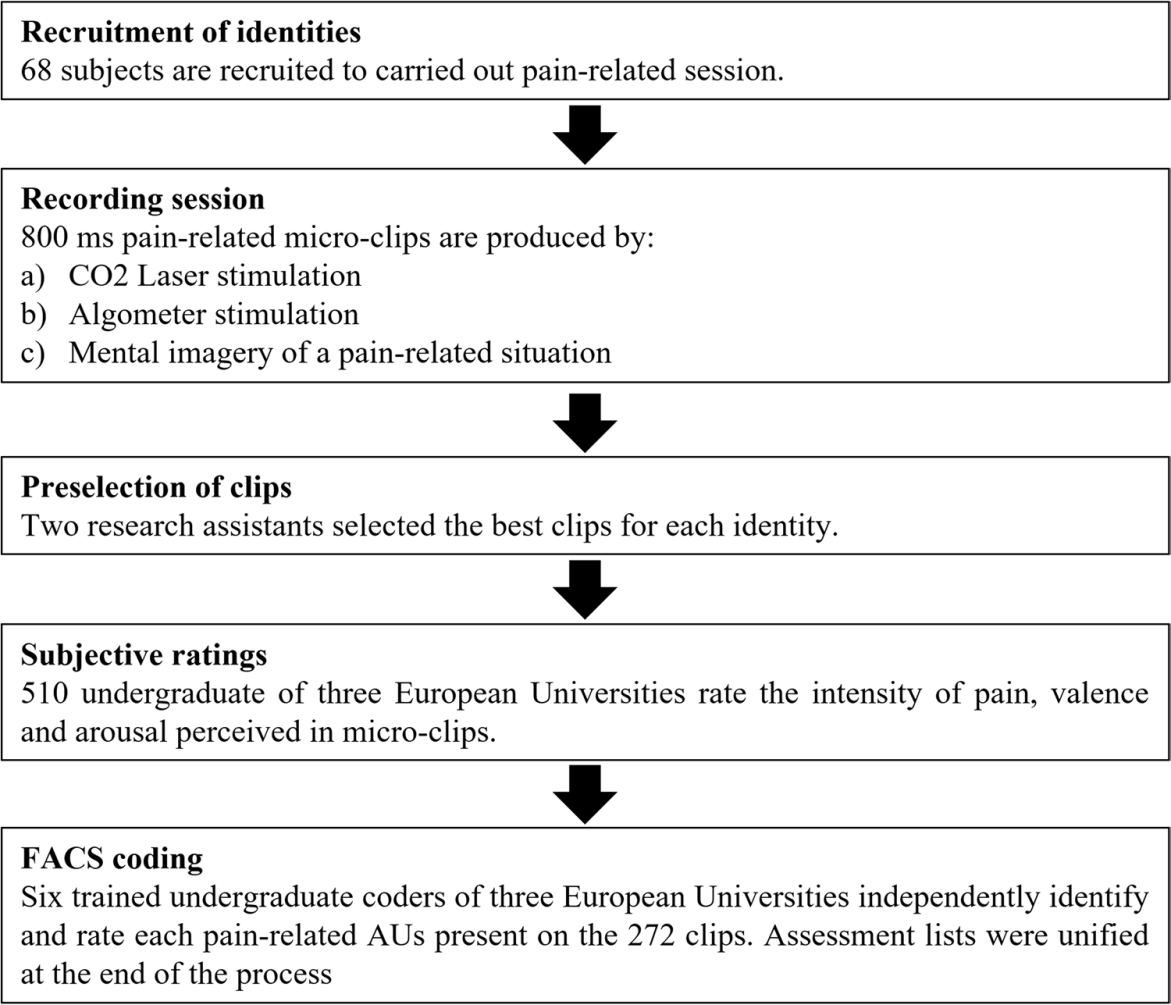
Diagram illustrating the workflow for the clip creation and validation method

Figure 1 illustrates the methodological sequence. Firstly, models were informed that painful stimulation was going to be applied through a CO_2_ laser system (Neurolas, Electronic Engineering; wavelength of 10.6 μm). CO_2_ laser parameters were set to a power of 9 watts and a duration of 30 ms. These configuration parameters of the intensity of painful stimulation were selected according to data provided by previous studies (see Peláez et al., 2019, for a more detailed description). Painful stimulation was delivered via a mean beam diameter of 4 mm (density = 21 mJ/mm^2^) over the dorsum of participants’ non-dominant hand. Second, a painful stimulation by pressure was applied on the index finger by means of an algometer for collecting the second type of pain-related facial expression. The algometer (Wagner, Force Dial™ FDK/FND Series) was a handheld device and had a 1 cm^2^ round rubber application surface. The device’s resolution was to 0.2 N, with 250 N capacity. Third, participants were asked to present a posed facial pain expression imagining a specific potentially painful situation (e.g., electric shock, headache, or cutting their finger). They were told that posed pain expressions should represent a feeling of pain ranging between 5 and 8 (“painful but you can bear it”) on a pain scale of 1 (absence of pain) to 10 (the worst pain imaginable). Finally, participants were instructed to present a neutral expression for one second. For the entire session, participants were told to keep their eyes directed to the camera and to try to avoid making sudden movements (turning away from the camera, touching their face with their hand, among others). A randomized procedure for filming session was carried out (i.e., CO_2_ laser, algometer, posed and neutral conditions were counterbalanced). Video sequences were segmented in epochs of 800 ms and were scanned in order to extract the facial clips with the most potent expression of pain.

**Fig. 2:**
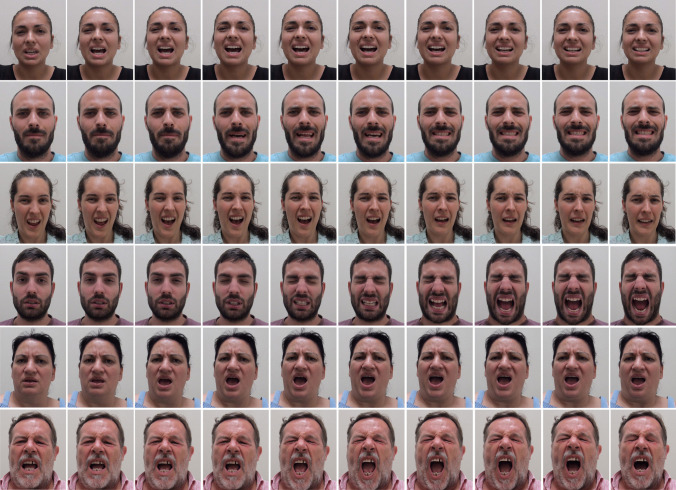
Examples of frames involved in pain-related faces. Figure reflects 10 of the 20 frames belonging to micro-clips

A total of 272 micro-clips were ultimately extracted (68 participants × 4 facial expressions: phasic spontaneous pain by CO_2_ laser, tonic spontaneous pain by algometer, posed pain and neutral). All micro-clips were 452 × 549 pixels. Luminosity and chromatic complexity were also calculated for each clip. The average luminosity of each video was computed using the Adobe Photoshop histogram tool (Adobe Systems Inc., 2020). To facilitate the use of both static and/or dynamic faces for experimental designs, the filming team extracted 20 frames for each micro-clip, which are also included in the PEMF (Fig. 2). Additionally, a version cropped to an oval shape (3.26 cm × 5.04 cm) was created for both pictures and micro-clips. Finally, a black and white version of both types of stimuli was also added. While PEMF describes and contains a diversity and variability of pain-related micro-clips, some research may require additional information regarding these stimuli (e.g., the use of new filters or croppers). In this sense, non-edited clips can also be downloaded by researchers interested in manipulating any parameters according to their own research interests. PEMF stimuli are currently available at both www.psicofis.wixsite.com/necodor/copia-de-enlaces and https://osf.io/3hgca/?view_only=12b04cd8164d4a6784c04b8c83bf95fb.

### Participants

A total of 510 undergraduate student volunteers (375 women and 135 men), between 17 and 50 years of age (mean = 20.37; SD = 3.95), participated in the micro-clip validation session. They were from three different European universities: (1) Rey Juan Carlos University (Madrid, Spain), (2) Complutense University of Madrid and (3) Maastricht University (Maastricht, Netherlands). These participants were recruited in their school universities, via either advertisement posted at each university or institutional email. No group differences were found in age, *F*_(3,508)_ = 1.772; *p* = 0.152, or educational level, *F*_(3,508)_ = 0.947; *p* = 0.418. Data for each student group are displayed in Table 1.

**Table 1:** Institution, number (male and female) and age (mean and SD) of participants, and micro-clips assessed in each evaluation

University	No. (male/female)	Mean age	No. clips
Rey Juan Carlos (Spain), set 1	75 (16/59)	21.2 (±4.6)	82/164*
Rey Juan Carlos (Spain), set 2	255 (47/208)	20.7 (±2.3)	108/108**
Complutense (Spain), set 1	100 (28/72)	20.7 (±3.6)	82/164*
Maastricht (Netherlands), set 1	80 (40/40)	21.6 (±2.5)	164/164*

All micro-clips were assessed for 255 students

*First set of 164 clips. **Remaining set of 108 (total number of micro-clips: 272)

### Procedure

Before data collection, all participants were informed of the objective of the study and that they were free to withdraw at any time. The rating process was divided into two time phases. The first set of 164 micro-clips was evaluated by 255 students in a random procedure distributed across the three European universities: 75 students from Rey Juan Carlos University, 100 from University Complutense of Madrid and 80 students from Maastricht University. The remaining 108 micro-clips (up to a total of 272 clips) were assessed by another independent sample of 255 Spanish students from Rey Juan Carlos University (see Table 1).

They were asked to rate micro-clips on a bidimensional scaling test (9-point Likert scales) for assessing intensity of pain (from 0, “no pain”, to 8, “greatest imaginable pain”), valence (from 0, “highly unpleasant”, to 8, “highly pleasant”), and arousal (from 0, “low arousal level”, to 8, “high level of arousal”). To assess whether facial expressions of pain displayed at each clip were spontaneous or posed, participants were instructed to respond to an additional question (“does the participant show a real pain face?”) via a dichotomous answer. Participants also had to make judgements about the presence of other basic emotions apart from pain (“do the facial expressions show any other emotion?”), through a checklist including six options (“happiness”, “sadness”, “anger”, “surprise”, “fear” or “disgust”). In other words, they had to select any other emotional expression they thought was represented in each clip as well. The instructions provided to the participants are available in the supplementary material (Appendix A1).

Instructions were always given in person so that participants could ask for further clarification if needed. This face-to-face format for the validation sessions avoids the distractibility caused by the absence of a responsible instructor, as may occur in online or self-administered procedures. For each session, micro-clips were displayed under optimal lighting and acoustic conditions through an individual Windows PC. Stimuli were presented and rated through Google forms at both Spanish universities and through Qualtrics at Maastricht University. In order to avoid possible anchoring effects, the order of micro-clips was randomized. Each clip was presented in loop format (i.e., animated GIF).

### Facial action coding system procedure

**Fig. 3:**
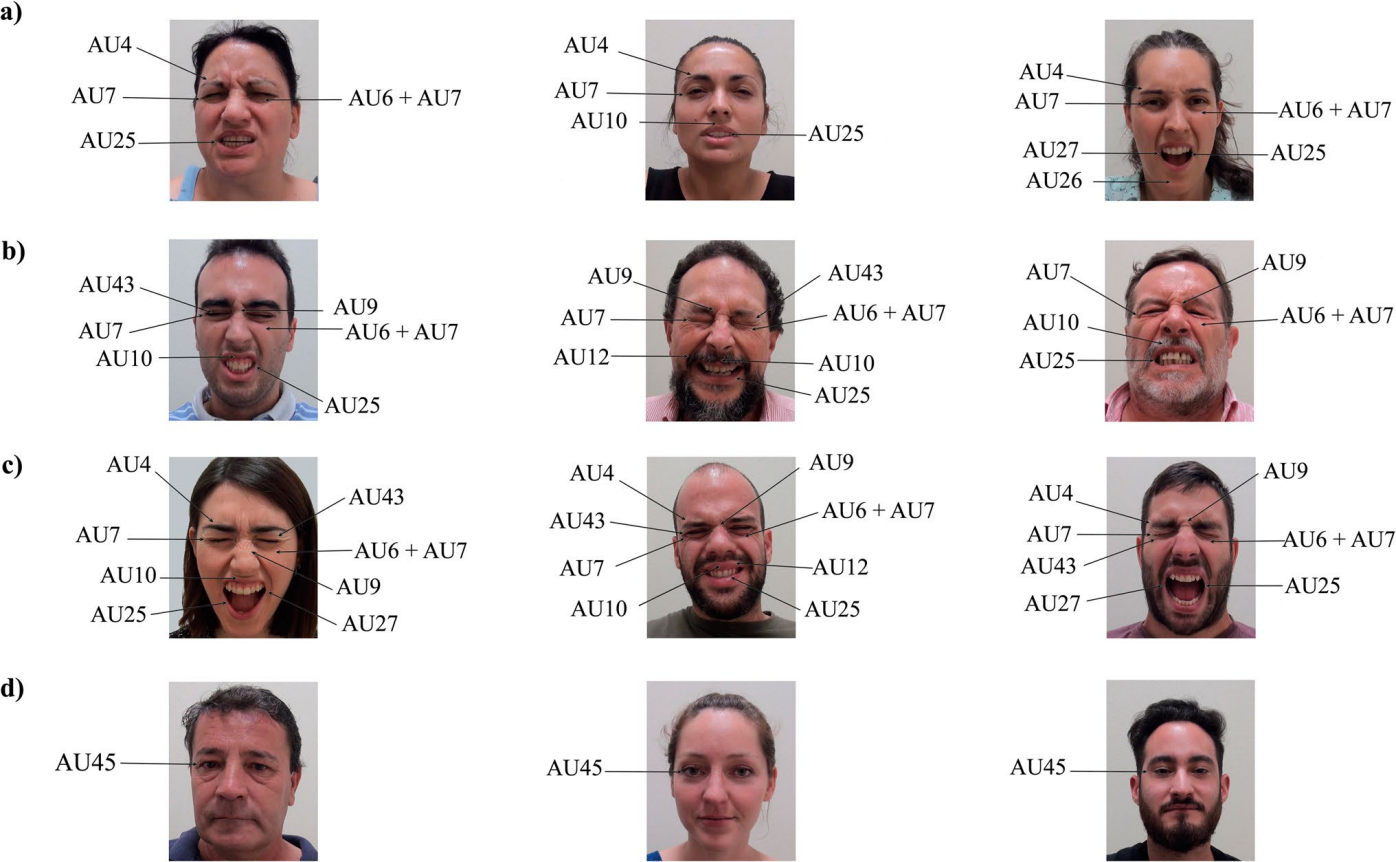
Examples of AUs in pain-related faces for each category: **a** algometer; **b** CO_2_ laser; **c** posed; **d** neutral

In order to provide sufficient characterization of facial micro-clips, six research assistants (hereinafter referred to as “coders”) were asked to code facial action units (AUs) using the Facial Action Coding System (Ekman et al., 2002). Coders were three women and three men aged 24–26 years (mean 25; SD 0.89). They were trained on the FACS system for one week, using the pain-related stimuli included in the Simon database (Simon et al., 2008). The experimenters provided them with a summary of the main pain-related AUs and a specific score sheet (see Appendix A2 and Appendix A3 in the supplementary material). During this coding procedure, coders extracted the occurrence of visible AUs in the different dynamic facial expressions. Each coder viewed all 272 videos (68 identities × 4 types of facial expressions: spontaneous—CO_2_ laser and algometer pain, posed and neutral) to determine the presence/absence of each specific pain-related AU (see Fig. 3). After this individual coding, commonly observed AU patterns were extracted. Every AU was considered representative of each facial expression as long as it was detected by at least four coders. Table 2 shows a brief description of pain-related AUs and the presence of common patterns (i.e., the ratio) that were detected on each type of facial expression (i.e., those where four or more independent coders agreed).

**Table 2:** Description of pain-related facial action units (AUs)

Action unit	Name (Description)	CO_2_ Laser	Algometer	Posed	Neutral
4	“Brow lowered”: The eye cover sinks and narrows the eye aperture. Eyebrows are pulled closer together and wrinkles between them may appear.	43.7 (43.2)	49.5 (36.6)	75.5 (24.9)	0.0 (0.0)
6	“Cheek raiser”: The upward elevation of the cheeks towards the eyes, thereby constricting the eye aperture and deepening the furrow below the eye.	33.1 (38.7)	63.3 (41.3)	64.2 (34.5)	0.5 (2.7)
7	“Lid tightener”: The eyelid is tightened and the lower eyelid rises.	41.2 (37.1)	48.7 (31.8)	71.0 (26.4)	0.0 (0.0)
9	“Nose wrinkler”: The nose becomes wrinkled, causing deepening of the nasolabial furrow which runs from both sides of the nose down to the sides of the mouth, as well as wrinkling of the infraorbital furrow, which is a triangle under the eye.	20.9 (31.4)	41.2 (40.1)	50.0 (38.4)	0.0 (0.0)
10	“Upper lip raiser”: Displays raising of the upper lip, leading to an angular shape of the upper lip. A deepening of the nasolabial and the infraorbital furrow is also visible.	33.4 (35.8)	45.5 (33.2)	38.2 (33.4)	0.2 (1.9)
12	“Lip corner puller”: The corners of the lips are pulled either upwards or downwards, resulting in a stronger infraorbital and nasolabial furrow.	25.0 (32.4)	32.5 (31.3)	38.0 (33.4)	1.7 (7.0)
20	“Lip stretcher”: An elongation of the lips whereby the lips become slimmer and the mouth stretches laterally.	28.9 (35.7)	32.3 (29.8)	31.6 (29.9)	1.9 (6.6)
25	“Mouth open”: Is active, the mouth opens, which may show the person’s teeth.	59.9 (44.9)	79.7 (30.8)	67.0 (39.2)	4.6 (13.7)
26	“Jaw drop”: A calm drop of the jaw whereby the teeth separate.	40.1 (41.4)	47.8 (33.3)	28.7 (31.4)	1.2 (5.1)
27	“Mouth stretch”: When the jaw is dropped forcefully and the mouth forms a vertical aperture action.	32.1 (38.3)	36.3 (38.7)	23.8 (35.6)	0.2 (1.9)
43	“Eyes closed”: Is characterized by a closure of the eye, revealing the upper eyelid extensively.	49.9 (37.8)	41.1 (37.4)	53.1 (31.3)	0.5 (2.7)
45	“Blink”: It is the opening and closing of eyes quickly before a stimulus onset.	34.2 (41.6)	20.7 (33.6)	24.3 (32.5)	78.8 (36.9)

Mean percentage and standard deviation (in parentheses) related to the presence of AU common patterns (i.e., those where four or more independent coders agreed) for each type of micro-clip

### Data analyses

As previously recommended and reported (Ruiz-Padial et al., 2021; Wierzba et al., 2015), the internal consistency of participant assessments was estimated by calculating split-half reliability scores. To this end, participants were numbered according to their order of participation. Each sample of participants who evaluated each of the two sets of micro-clips (i.e., 255; see Table 1) was split into two subgroups according to a random procedure and dividing the sample as a function of the gender of the participants. The average ratings for intensity of pain, valence and arousal were then calculated separately for each individual micro-clip and within each participant subgroup. Finally, Pearson correlations among these average ratings were computed for each of the two subgroups of participants of each sample.

Next, we analysed whether the ratings of intensity of pain, valence and arousal were different for each type of micro-clip (i.e., phasic spontaneous CO_2_ laser, tonic spontaneous algometer, posed pain and neutral) through a series of repeated-measures analyses of variance (ANOVAs). Potential differences in these variables as a function of the model’s gender and age, as well as the participant gender, were also tested through repeated-measures ANOVAs. In all contrasts described in this section, Greenhouse–Geisser (GG) epsilon correction was applied to adjust degrees of freedom of the *F* statistic. Effect sizes were computed through the eta-square (η^2^_p_) technique. Post hoc comparisons were made to determine the significance of pairwise contrast, using the Bonferroni test (alpha = .05). Additionally, as previously recommended (Brysbaert, 2019), effect sizes of pairwise contrasts were determined by Cohen’s *d* (Cohen, 2013). All statistical analyses described in this section were performed using SPSS Statistics 25 (IBM Corporation, Armonk, NY, USA). To minimize the possible effects of biased responses, an outlier checking procedure was performed. Individual ratings that deviated more than 2 SD from the intensity of pain, valence and arousal average were removed from analyses.

BOMThe ability of participants to discriminate the veracity of pain-related facial expressions (i.e., spontaneous vs posed) was also explored. Repeated-measures ANOVAs were computed to compare the ratio of responses in which participants considered that micro-clips represented a real expression across the different types of facial expressions. The presence of other discrete emotions (i.e., happiness, fear, disgust, surprise, anger and sadness) conveyed by each facial expression (i.e., CO_2_ laser, algometer, posed pain and neutral) was also compared through repeated-measures ANOVAs.

Regarding the ratings of FACS codes given by coders, a series of statistical analyses were also performed. First, we extracted the number of responses given by coders who checked the occurrence of visible AUs in the different dynamic facial expressions (see supplementary material: Table S1). Additionally, we computed the presence of common patterns (i.e., the ratio) that were detected on each type of face (i.e., those where four or more independent coders agreed). Second, in order to detect possible differences in these common patterns of AU ratios for micro-clips for each facial expression (i.e., CO_2_ laser, algometer, posed pain and neutral), repeated-measures ANOVAs were applied. And third, potential differences in pain-related AU ratios were tested as a function of models’ gender (male or female) through repeated-measures ANOVAs.

Finally, the relationships between dimensional ratings of facial expressions (i.e., intensity of pain, valence, and arousal) and the total number of pain-related AUs provided by coders in the whole set of micro-clips were examined by computation of bivariate Pearson correlations.

## Results

Subjective ratings on intensity of pain, valence and arousal, as well as descriptive data for the entire set of facial expressions (i.e., type of micro-clip, label, luminosity, gender, age, percentage of agreements for other discrete emotions and the presence of representative pain-related AUs), are summarized in the supplementary material (Table S1).

### Reliability

As mentioned previously, the internal consistency of participant assessments was estimated by calculating split-half reliability scores. All Pearson correlations were significant (*p* < 0.001), and Spearman-Brown-corrected reliability scores were particularly high for the two sets of micro-clips in the dimensional ratings of intensity of pain, *r* = 0.99, set 1; *r* = 0.99, set 2; valence, *r* = 0.98, set 1; *r* = 0.99, set 2; and arousal, *r* = 0.99, set 1; *r* = 0.99, set 2. Similarly, Spearman-Brown-corrected scores on gender split-half reliability were high for the two sets of micro-clips in the dimensional ratings of intensity of pain, *r* = 0.98, set 1; *r* = 0.97, set 2; valence, *r* = 0.97, set 1; *r* = 0.95, set 2; and arousal, *r* = 0.97, set 1; *r* = 0.97, set 2. Therefore, dimensional rating provided by the different groups of participants might be considered highly homogeneous.

## Dimensional ratings: Intensity of pain, valence and arousal

**Fig. 4:**
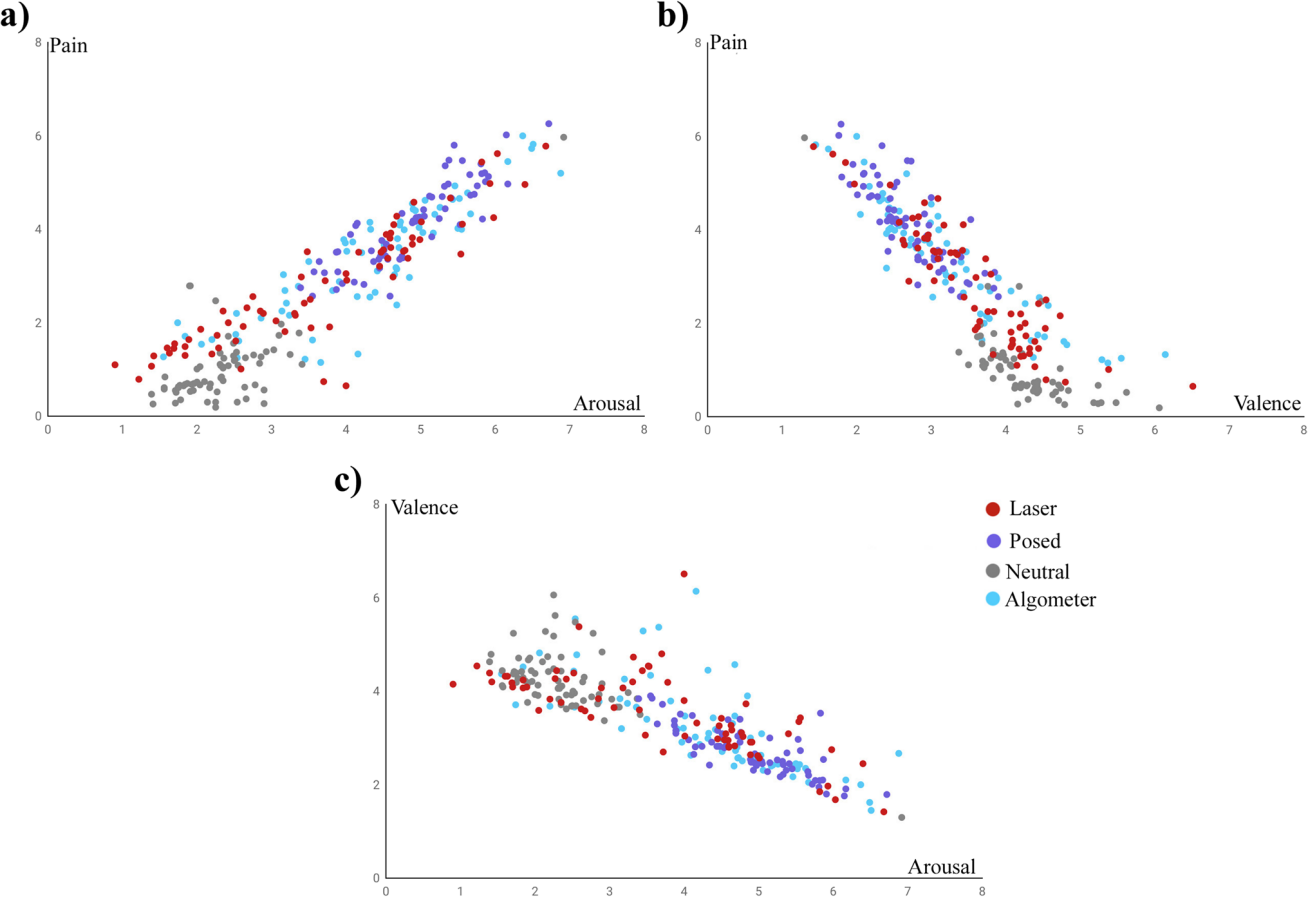
Scatterplot representing average (**a**) pain intensity **×** arousal, (**b**) pain intensity **×** valence and (**c**) valence **×** arousal, provided in each picture by the experimental samples

PEMF provides interactive scatterplots (see www.psicofis.wixsite.com/necodor/copia-de-enlaces) where researchers can check the space location of each micro-clip considering the relationships of the three dimensional variables: (1) intensity of pain × valence space, (2) intensity of pain × arousal space and (3) valence × arousal space. Scatterplots allow for rapid visual selection of micro-clips according to their values in such dimensions (see Fig. 4).

ANOVAs related to the effect of micro-clip types on subjective dimensional ratings yielded significant differences for intensity of pain, *F*_(1,67)_ = 149.4; *p* < 0.001; η^2^_p_ = 0.69, valence, *F*_(1,67)_ = 61.7; *p* < 0.001; η^2^_p_ = 0.48, and arousal, *F*_(1,67)_ = 104.0; *p* < 0.001; η^2^_p_ = 0.61. As expected, ratings of pain-related expressions showed higher intensity of pain, lower valence and higher arousal scores than those of faces that did not display pain (*p* < 0.001). Post hoc contrasts revealed that posed pain-related faces showed higher intensity of pain, more negative valence and higher arousal values than spontaneous pain-related faces (both CO_2_ laser and algometer) (*p* < 0.01). Interestingly, differences between spontaneous pain-related facial expressions occurred only for intensity of pain and arousal ratings, where pain-related faces elicited by algometer showed higher pain intensity (*p* = 0.012) and higher arousal values (*p* = 0.003) than CO_2_ laser pain-related faces. Table 3 shows average scores of pain intensity, valence and arousal associated with each type of micro-clip. Table 4 shows effect sizes of pairwise contrasts (Cohen’s *d*) related to pain intensity, valence and arousal for each type of micro-clip.

**Table 3:** Means and SD of intensity, valence and arousal of each type of micro-clip category by gender of micro-clip

Type of pain	Intensity			Valence			Arousal		
	Total	Women	Men	Total	Women	Men	Total	Women	Men
CO_2_ laser Algometer Posed	2.8 (1.3) 3.3 (1.2) 4.1 (0.9)	2.9 (1.2) 3.4 (1.2) 4.1 (0.8)	2.6 (1.3) 3.2 (1.2) 4.3 (0.9)	3.5 (0.8) 3.2 (0.9) 2.7 (0.5)	3.4 (0.9) 3.2 (0.9) 2.8 (0.5)	3.6 (0.6) 3.2 (1.0) 2.6 (0.5)	3.7 (1.4) 4.2 (1.2) 4.9 (0.7)	3.9 (1.2) 4.3 (1.2) 4.8 (0.8)	3.3 (1.6) 4.1 (1.2) 5.1 (0.8)
Neutral	0.9 (0.5)	0.8 (0.4)	1.1 (0.7)	4.2 (0.5)	4.3 (0.5)	4.1 (0.4)	2.2 (0.4)	2.2 (0.5)	2.3 (0.5)

**Table 4:** Effect sizes (Cohen’s *d*) of pairwise contrasts relative to pain intensity, valence and arousal

Pairwise contrast	Intensity	Valence	Arousal
Posed × Neutral Posed × CO_2_ laser Posed × Algometer	4.2^**^ 1.1^**^ 0.7^**^	2.9^**^ 1.1^**^ 0.7^**^	4.1^**^ 1.1^**^ 0.6^**^
Algometer × Neutral	2.5^**^	1.3^**^	2.2^**^
Algometer × CO_2_ laser	0.4^*^	0.3	0.4^**^
CO_2_ laser × Neutral	1.9^**^	1.3^**^	1.3^**^

*p < 0.05, **p < 0.01

### Analyses for discriminating the veracity of pain-related facial expressions

ANOVAs showed statistical differences for recognizing the veracity expressed by each type of pain-related faces, *F*_(1,67)_ = 5.1; *p* = 0.007; η^2^_p_ = 0.05. Post hoc analyses revealed that the ratio of participants’ responses that considered spontaneous pain-related faces as real (CO_2_ laser: mean = 56.4%; SD = 13.53; algometer: mean = 57.1%; SD = 15.27) was lower than those who rated them as posed pain faces **(**mean = 63%; SD = 13.80; CO_2_ laser vs posed: *p* = 0.01, *d* = 0.48; algometer vs posed: *p* = 0.02, *d* = 0.40). The comparison between types of spontaneous expressions, CO_2_ laser and algometer, failed to show significant differences (*p* > 0.05). In brief, posed pain expressions were recognized as real in a higher proportion than spontaneous pain faces.

### Discrete ratings: Basic emotions

As described in the Introduction section, we expected that not all facial expressions included in PEMF would be exclusively perceived as painful. In this sense, we explored the presence of other discrete emotions (see subjective ratings procedure). Disgust, *F*_(1,67)_ = 39.8; *p* < 0.001; η^2^_p_ = 0.37, fear, *F*_(1,67)_ = 39.9; *p* < 0.001; η^2^_p_ = 0.36, and surprise, *F*_(1,67)_ = 19.5; *p* < 0.001; η^2^_p_ = 0.23, were differently represented among pain-related faces. Specifically, post hoc contrasts showed that the average number of participants detecting disgust in pain-related micro-clips was higher for algometer (*p* < 0.001, *d* = 1.19) and posed pain expressions (*p* < 0.001, *d* = 1.83) than for CO_2_ laser facial expressions. The pattern describing the presence of surprise in pain-related faces was the opposite (i.e., higher mean values in CO_2_ laser than in both algometer, *p* = 0.05, *d* = 0.42, and posed faces, *p* = 0.03, *d* = 0.47). Finally, posed pain-related faces showed higher mean values for fear than both algometer (*p* < 0.001, *d* = 0.80) and CO_2_ laser spontaneous faces (*p* < 0.001, *d* = 1.08). In contrast, ANOVAs showed no significant differences for happiness, *F*_(1,67)_ = 2.3; *p* = 0.054. A full description of the ratio of discrete emotions for each type of micro-clip can be seen in Table 5.

**Table 5:** Mean percentage and SD of participants’ ratings of basic emotions for each type of micro-clip

Type of pain	Disgust	Fear	Sadness	Surprise	Happiness	Anger	Any
CO_2_ laser Algometer	8.8 (10.1) 16.2 (12.8)	8.2 (6.4) 9.8 (8.1)	4.6 (5.8) 5.2 (5.6)	25.7 (20.2) 18.6 (11.4)	9.9 (16.3) 11.1 (18.6)	7.3 (7.6) 10.4 (11.1)	35.2 (22.3) 28.5 (11.1)
Posed	20.1 (10.9)	16.6 (8.6)	6.7 (7.6)	17.8 (11.8)	5.1 (8.1)	8.3 (7.9)	25.1 (9.1)
Neutral	4.5 (4.8)	4.7 (3.9)	11.5 (8.4)	8.1 (8.5)	10.8 (15.4)	3.9 (4.8)	56.3 (14.1)

### Pain-related AU frequencies

The mean percentage of pain-related AUs detected in each facial expression is summarized in Table 2. ANOVAs showed that pain-related AUs were differently represented according to each type of facial expression. Such differences were found for AU4 “brow lowered”, *F*_(1,67)_ = 77.2; *p* < 0.001; η^2^_p_ = 0.53, AU6 “cheek raiser”, *F*_(1,67)_ = 62.4; *p* < 0.001; η^2^_p_ = 0.48, AU7 “lid tightener”, *F*_(1,67)_ = 87.6; *p* < 0.001; η^2^_p_ = 0.56, AU9 “nose wrinkler”, *F*_(1,67)_ = 41.6; *p* < 0.001; η^2^_p_ = 0.38, AU25 “mouth open”, *F*_(1,67)_ = 80.5; *p* < 0.001; η^2^_p_ = 0.54, and AU26 “jaw drop” codes, *F*_(1,67)_ = 31.7; *p* < 0.001; η^2^_p_ = 0.32. Specifically, AU4 and AU7 were highly presented in posed pain-related faces compared with both CO_2_ laser (*p* < 0.001, *d* = 0.89; *p* < 0.001, *d* = 0.92, respectively) and algometer spontaneous faces (*p* < 0.001, *d* = 0.82; *p* < 0.001, *d* = 0.92, respectively). Moreover, AU6 and AU9 were less frequently detected in CO_2_ laser faces compared to both posed (*p* < 0.001, *d* = 0.84; *p* < 0.001, *d* = 0.82, respectively) and algometer faces (*p* < 0.001, *d* = 0.75; *p* < 0.001, *d* = 0.56, respectively). Finally, facial expressions elicited by algometer were highly detected compared with CO_2_ laser faces for AU25 (*p* = 0.002; *d* = 0.36), and highly detected for AU26 compared with posed faces (*p* = 0.002, *d* = 0.58). In addition, analyses showed the presence of a higher number of pain-related AUs for all pain-related facial expressions (spontaneous and posed faces) as compared with neutral faces (*p* < 0.001).

### Gender and age differences

Potential interaction effects between the gender of micro-clips (model: male or female) by type of facial expression (CO_2_ laser, algometer, posed pain and neutral) were also examined for dimensional measures of pain intensity, valence and arousal. Differences did not reach statistical significance for any of the tested variables (intensity of pain, *F*_(1,67)_ = 1.6; *p* = 0.18, valence, *F*_(1,67)_ = 1.1; *p* = 0.36, and arousal, *F*_(1,67)_ = 2.5; *p* = 0.07. Table 3 displays averages of intensity of pain, valence and arousal by micro-clip gender. Similarly, the effect of the interaction between the gender of the participant and gender of the micro-clips showed no significant effects in intensity of pain, *F*_(1,67)_ = 1.4; *p* = 0.23, or arousal, *F*_(1,67)_ = 0.6; *p* = 0.41, scores. However, valence scores showed significant interaction effects, *F*_(1,67)_ = 49.5; *p* < 0.001; η^2^_p_ = 0.42. Post hoc contrasts revealed higher valence scores in female micro-clips when they were assessed by women than the values provided by male participants (*p* < 0.001, *d* = 0.42).

The effect of age of micro-clips (model: adult or elderly) by type of facial expression on intensity, valence and arousal scores did not yield statistical significance^1^. Similarly, the interaction between the gender of models by type of pain-related expressions on pain-related AUs, *F*_(1,67)_ = 2.436; *p* = 0.073, did not yield statistical significance.

### Correlations between intensity of pain, valence, arousal and action units (AUs)

Bivariate Pearson correlations were computed between dimensional ratings of facial expressions (i.e., intensity of pain, valence, and arousal) and the total number of pain-related AUs provided by coders in all micro-clips. These analyses revealed that the number of AUs was significantly associated with the intensity of pain, *r* = 0.71, *p* < 0.001, and arousal scores, *r* = 0.68, *p* < 0.001, presenting a linear association with positive slope: the higher the number of AUs, the greater the intensity of pain and arousal. Similarly, the number of AUs was significantly associated with the valence scores, *r* = −0.51, *p* < 0.001, and its linear association showed a negative slope: the higher the former, the lower the latter (more negative valence). Moreover, emotional valence was significantly associated with the intensity of pain, *r* = −0.89, *p* < 0.001, and arousal scores, *r* = 0.81, *p* < 0.001, also following a negative slope. Finally, intensity of pain and arousal were highly and positively associated, *r* = 0.92, *p* < 0.001.

## Discussion

Exploring pain processing usually requires a long set of standardized stimuli (Mende-Siedlecki et al., 2020). In this vein, a limited number of pain-related faces databases have been developed so far that are valuable tools for researchers in this scientific field (Lucey et al., 2011; Mende-Siedlecki et al., 2020; Simon et al., 2008; Zhang et al., 2014). Despite the usefulness of such databases, the creation of new sets of facial stimuli to fill some existing gaps, such as lack of age diversity, small samples or the absence of pain-related spontaneous expressions (statics and dynamics), might benefit pain research. Therefore, we developed the Pain E-Motion Faces Database (PEMF), a new pain database that includes dynamic micro-clips and static pictures depicting pain-related and matched neutral facial expressions. All stimuli have been rated on intensity of pain, valence and arousal dimensions, as well as on the presence of other discrete emotions (i.e., disgust, fear, sadness, surprise, happiness and anger). Pain-related action units (as provided by the FACS coding system; Ekman & Friesen, 1977) associated with each face are also reported. PEMF is the first database which provides both posed and spontaneous pain-related faces (in two modalities: tonic and phasic) from a large sample of non-actor participants ranging from 18 to 67 years of age. Additionally, the physical properties of micro-clips are reported in order to provide complementary information that can be helpful for the selection of pain-related facial expressions (clips and/or pictures) to develop future research designs. All micro-clips were created from scratch, being carefully chosen to be adaptable for contemporary canons.

As expected, pain-related faces showed higher intensity of pain, more negative valence and higher arousal than neutral facial expressions. Regarding the type of painful stimulation, tonic pain-related faces (i.e., algometer) showed higher intensity and arousal scores than phasic pain-related faces (i.e., CO_2_ laser). Importantly, this study confirmed that posed pain expressions were not interpreted as (and are not representative of) spontaneous pain expressions. Thus, posed pain-related faces showed higher intensity of pain, more negative valence and higher arousal than those representing spontaneous expressions of pain. Despite the fact that participants were not able to distinguish between spontaneous and posed pain-related facial expressions, the results revealed a statistical difference in the dimensional ratings, being more extreme for posed pain-related faces. In this sense, perceivers generally act randomly when distinguishing between real and posed pain (Littlewort et al., 2007; Littlewort et al., 2009; Poole & Craig, 1992). It has been argued that the recognition of real pain states by humans is not entirely accurate (Bartlett et al., 2014), even using computerized machine learning procedures (Littlewort et al., 2009). Moreover, some previous research has noted that posed emotions may result in more extreme expressions (Pantic & Rothkrantz, 2003; Zhihong Zeng et al., 2009), which can artificially increase the accuracy of their identification (Faso et al., 2015; Russell, 1997). Thus, posed expressions may not be valid analogues of the expressions that are produced when emotions are actually aroused, because they may include extraneous muscle movements, not including muscle movements that do appear spontaneously, and/or producing levels of intensity or asymmetries representative of spontaneous expressions (Matsumoto et al., 2009). However, when the feeling of pain is severe, spontaneous and simulated responses can be equivalent in intensity (Prkachin, 1992). Moreover, given their controlled nature and ease of identification (Faso et al., 2015; Matsumoto et al., 2009; Russell, 1997), facial expression databases usually consist of posed stimuli (Ekman, 1976; Georghiades et al., 2001; Goeleven et al., 2008; Mende-Siedlecki et al., 2020). Therefore, studies will benefit from databases that include both posed and spontaneous expressions, providing contexts closer to real-world functioning.

On the other hand, the present results showed significant differences in the ratio of participants indicating that basic emotions were distinctly represented in each type of micro-clip. Specifically, algometer and posed pain-related faces showed higher values of disgust than neutral and CO_2_ laser facial expressions. Moreover, posed pain-related faces showed higher values of fear compared with the rest of the facial expressions. Finally, CO_2_ laser-evoked facial expressions showed higher values of surprise than the other faces. Despite considering that the facial movements of pain-related faces have specific patterns (Prkachin, 1992; Prkachin & Solomon, 2008), there is some evidence suggesting the presence of overlapping patterns between pain-related expressions and other basic emotions such as fear, anger, sadness, disgust and surprise (Cordaro et al., 2018; Prkachin & Craig, 1995; Simon et al., 2008). However, the degree of overlap between pain expression and other basic emotions seems to be small (Cordaro et al., 2018; LeResche, 1982). This fact suggests that although such negative emotions may occur in conjunction with pain, or in reaction to the expression of pain, it is the unpleasant emotional component of the pain-related facial expression that is manifested in the face (LeResche, 1982). That is, the unpleasant emotional component of pain itself differs behaviourally from these emotions, and may also differ at both the experiential and physiological levels (Benuzzi et al., 2008; Cordaro et al., 2018; LeResche & Dworkin, 1988).

As expected, the analysis of basic constituents of each emotional expression revealed that neutral faces showed statistical differences (i.e., lower values) from the three types of pain-related expressions (i.e., real and posed pain-related faces) for all statistical AU contrasts. Moreover, in pain-related comparisons, posed pain-related faces showed higher agreements than spontaneous pain-related faces for AU4 (“brow lowered”) and AU7 (“lid tightened”). Moreover, CO_2_ laser pain-related faces showed lower agreements compared to algometer and posed pain-related faces for AU6 (“cheek raiser”) and AU9 (“nose wrinkler”). Similarly, CO_2_ laser faces showed lower agreements than posed faces for AU12 (“lip corner puller”) and lower agreement than algometer pain-related faces for AU25 (“mouth open”). Finally, algometer faces showed higher agreement compared only with posed faces for AU26 (“jaw drop”). As previously reported, four AUs have been strongly related across different pain modalities to pain-related faces (Prkachin, 1992; Prkachin & Solomon, 2008), although up to a total of 16 facial actions units have been detected in pain states (Williams, 2002). Some authors have reported that the distribution of facial movements (i.e., AUs) varies from one type of painful stimulation to another (Prkachin, 1992). For instance, AU4 (“brow lowering”), AU12 (“lip corner puller”) and AU43 (“eyes closed”) have been related to more intense values for tonic pain-related faces (i.e., electric shock) than the other pain modalities. Other investigations have reported, however, that lowering the brows (AU4), cheek raise/lid tightening (AUs 6, 7), nose wrinkling/raising the upper lip (AUs 9, 10) and opening of the mouth (AUs 25, 26, 27) appear independently of the cognitive state of individuals and remain stable in the presence of both clinical and experimental pain (Kunz et al., 2019).

Another interesting question explored in the present study was related to the potential effect of the model’s gender on the assessments given for each type of facial expression. This research found no significant differences among expressions. Although some previous studies have shown that women tend to be more emotionally expressive than men (Buck et al., 1974; Keogh, 2014; Kring & Gordon, 1998; LaFrance et al., 2003), the evidence of gender bias in the perception of pain-related states is mixed and scarce (Craig et al., 1991; Guinsburg et al., 2000; Keogh, 2014; Prkachin, 1992). While some data showed gender differences in pain perception in a sample of children (Guinsburg et al., 2000), results in adults are quite inconsistent (Kunz et al., 2006; Prkachin, 1992; Simon et al., 2008; Vlaeyen et al., 2009). The same expression of pain may be differently interpreted as a function of the observer’s gender. In particular, women’s expressions were considered to represent greater pain intensity and negative mood (Hirsh et al., 2008). However, other studies found no gender differences when participants evaluated pain-related expressions in response to tonic heat stimulation (Kunz et al., 2006).

Nevertheless, the current study has some limitations that should be considered in future investigations. Despite the large sample size, the entire sample of models comprises Caucasian individuals, which makes it difficult to use it in cross-cultural investigations of painful expressions. Additionally, participants who took part in the micro-clip validation session were quite homogeneous in some demographic variables, such as age and education (the whole sample of participants were university students). These facts might limit the generalization of the current results. Nevertheless, we will continue to expand the PEMF database by recruiting new identities to minimize such limitations.

Based on the results obtained, PEMF can be considered a useful tool that might allow researchers to examine behavioural and neural mechanisms related to cognitive and affective processing in different contexts, such as that which occurs in chronic pain patients (Fernandes-Magalhaes et al., 2022). On the other hand, PEMF could be useful in the study of pain judgments in clinical contexts, such as psychology, neurology or psychiatry. Moreover, the use of dynamic stimuli could enable the emotional reaction to be intensified, allow for better recognition of facial expression and evoke more intense reactions in the viewer than static faces. Finally, it is recommended that the results discussed here are considered for selecting pain-related facial expression micro-clips to conduct future experimental studies.

### Supplementary Information

ESM 1 (DOCX 258 kb)

## Data Availability

The data used for this article (ratings and physical properties of each image) are available as online supplementary material: https://osf.io/3hgca/?view_only=12b04cd8164d4a6784c04b8c83bf95fb.

## Footnotes

1 To explore the possibility that intensity, valence and arousal score effects might be modulated by the age of models, potential interaction effects of the age of micro-clips (model: adult or elderly) by type of facial expression (CO_2_ laser, algometer, posed pain and neutral) were also examined for these dimensional measures. Differences did not reach statistical significance for any of the tested variables (intensity of pain, *F*_(1,67)_ = 0.6; *p* = 0.58; valence, *F*_(1,67)_ = 0.2; *p* = 0.90; and arousal, *F*_(1,67)_ = 0.8; *p* = 0.46.
