# Supplementary material for: Pain E-motion Faces Database (PEMF): Pain-related micro-clips for emotion research
Source: Behav Res Methods. 2022 Oct 17;55(7):3831–44. doi: 10.3758/s13428-022-01992-4 (PMC10615976; doi:10.3758/s13428-022-01992-4)
Supplement: Supplementary file 1 — (DOCX 258 kb) [file 13428_2022_1992_MOESM1_ESM.docx]

**Supplementary materials for “Pain E-motion Faces Database (PEMF): pain-related micro-clips for emotional research”**

Roberto Fernandes-Magalhaes, Alberto Carpio, David Ferrera, Dimitri Van Ryckeghem, Irene Peláez, Paloma Barjola , María Eugenia De lahoz, María Carmen Martín-Buro, J. Antonio Hinojosa, Stefaan Van Damme, Luis Carretié, Francisco Mercado

**Appendix A1: Instructions provided to participants questionaire**

**Instructions:** “Thank you for completing the first part of the session. Now, you can start the session. During this part you will view short micro-clips of facial expressions. Your task will consist to carefully attend to these micro-clips and rate them on several dimensions. You will be able to watch the micro-clips more than once, but it is not necessary to spend a lot of time on each clip. The first impression is the important one. Responses will be treated anonymously. If anything is unclear, please ask the experimenter. During this session, you can take a break if you need.”

**Question 1:** What is your Gender?

- Female
- Male

**Question 2:** What is your Age?

______________________________________________________________________

**Question 3:** What is your Nationality?

_­­­­­­­­­­­_____________________________________________________________________

**Micro-clip presentation**


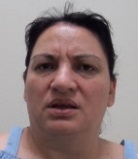

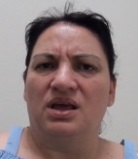

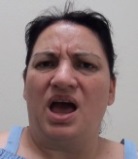

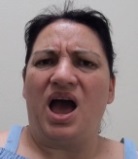

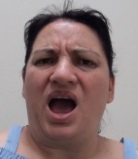

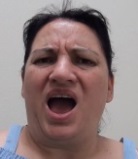

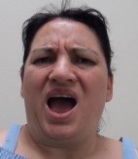

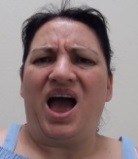

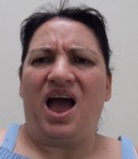

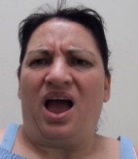


**Question 4:** Please rate the intensity of pain in the micro-clip.

(Not at all intense) 0 1 2 3 4 5 6 7 8 (Most intense possible)

**Question 5:** Please rate how the person in the clip might feel, with respect to arousal.

(Highly relaxed) 0 1 2 3 4 5 6 7 8 (High level of arousal)

**Question 6:** Please rate how the person in the clip might feel, with respect to valence.

(Extremely unpleasant) 0 1 2 3 4 5 6 7 8 (Extremely pleasant)

**Question 7:** Does the participant show a real pain face?

- Yes
- No

**Question 8:** Do the facial expressions show any other emotion? (You can choose multiple options or choose nothing if you don't detect any other emotions)

- Happiness
- Sadness
- Anger
- Surprise
- Fear
- Disgust

**Appendix A2: Score Sheet AUs**

**Instructions:** “Thank you for completing this part of the session. During this part of the session you will view short micro-clips of facial expressions. Your task is to attend to these micro-clips and rate each video clip on several AUs. A brief summary of the main pain-related AUs is available to check. You will be able to watch the micro-clips more than once. Responses will be treated anonymously. During this session, you can take a break if you need one.”

**Pain-related facial action coding system summary**

**Brow Lowered (AU4):**

- Lowers the eyebrow: sometimes the inner part of the eyebrow or the entire eyebrow may appear to be lowered.
- Reduce the eye opening.
- Brings the eyebrows closer together.
- Creases between the eyebrows
- An oblique wrinkle or muscle bulge is produced from the middle of the forehead to below the eyebrow.

**Cheek Raiser and Lid Compressor (AU6):**

- Pull the skin towards the eye from the cheeks and the outer musculature of the eye contracts.
- Lifts the cheek upwards.
- The eye opening narrows.
- Crow's feet wrinkles appear at the corner of the eye.
- Deepens the lower eye lid.
- Lateral portion of eyebrows lowered.
- If AU 6 is strong it can:
  - Deepen the nasolabial fold or lift the outer portions of the upper lip.

**Lid Tightened (Au7):**

- Tightens the eyelids.
- Narrows the eye opening.
- Lifts the lower eyelid, which may be straight or curved.

**Nose Wrinkled (AU9):**

- Wrinkles appear on the nose.
- The skin is bunched up near the lower eyelid.
- It lowers the medial portion of the eyebrows, hiding the elevation of the inner eyebrows.
- The eye opening is reduced.
- The upper lip is elevated and may separate.
- The nose is widened.
- Deepens the nasolabial fold.

**Upper Lip Raiser (AU10):**

- Raises the upper lip.
- Angular curve in the upper lip.
- Deepens the infraorbital sulcus.
- Deepens and lifts the nasolabial fold.
- Widens and lifts the wings of the nose.
- If the action is strong, the lips separate.

**Lip Corner Puller (AU12):**

- The corners of the lips backwards and upwards.
- Deepens the nasolabial fold, pulling it upwards.
- In a moderate AU12, the infraorbital sulcus is deepened.
- In a strong AU12:
  - Infraorbital deepening is more evident.
  - Reduces the eye opening
  - Produces crow's feet
  - May lift and widen the nose
- Note the combinations of 6 + 12, 7 + 12 and 6 + 7.

**Lip Stretcher (AU20):**

- Pull the lips back laterally, the main movement of the lips is horizontal.
- Lengthens the mouth
- Lips are flattened and stretched laterally
- Cheek near the lips flattens out
- Wrinkles may appear at the corners of the lips, this is not so important.
- The nasolabial fold is stretched.
- The chin may appear wrinkled or flattened.
- The nostril may lengthen

**Lips Part (AU25):**

- Teeth and gums can be exposed

**Jaw Drop (AU26):**

- The jaw is lowered, and separation of the teeth can be inferred.
- A space between the teeth can be seen if the lips are parted. (25 + 26)

**Mouth Stretch (AU27):**

- Jaw pulls downwards
- The mouth can be opened in the shape of an oval.
- Lips can be stretched vertically according to the extension of the mouth.
- Cheeks are flattened and stretched

**Eye Closure (AU43):**

- The eye opening is reduced.
- The upper part of the eye is exposed.
- If there are signs of eyelid tension, a score of 6 or 7 is given.

**Blink (AU45):**

- The eyes open and close very quickly and without pause.
- Should not last more than half a second
- Must be bilateral

| Clip | Nº | Sex | Brow Lowered (AU4) | Cheek Raiser and Lid Compressor (AU6) | Lid Tightened (AU7) | Nose Wrinkled (AU9) | Upper Lip Raiser (AU10) | Lip Corner Puller (AU12) | Lip Stretcher (AU20) | Lips Part (AU25) | Jaw Drop (AU26) | Mouth Stretch (AU27) | Eye Closure (AU43) | Blink (AU45) |
| --- | --- | --- | --- | --- | --- | --- | --- | --- | --- | --- | --- | --- | --- | --- |
| S001 | 1 | F |  | x | x | x |  |  |  | x |  | x |  |  |
|  | 2 | F |  |  |  |  |  | x |  |  |  |  |  |  |
|  | 3 | F |  | x | x |  |  |  |  | x |  |  |  |  |
|  | 4 | F |  |  |  |  |  |  |  |  |  |  |  | x |
| S002 | 1 |  |  |  |  |  |  |  |  |  |  |  |  |  |
|  | 2 |  |  |  |  |  |  |  |  |  |  |  |  |  |
|  | 3 |  |  |  |  |  |  |  |  |  |  |  |  |  |
|  | 4 |  |  |  |  |  |  |  |  |  |  |  |  |  |
| S003 | 1 |  |  |  |  |  |  |  |  |  |  |  |  |  |
|  | 2 |  |  |  |  |  |  |  |  |  |  |  |  |  |
|  | 3 |  |  |  |  |  |  |  |  |  |  |  |  |  |
|  | 4 |  |  |  |  |  |  |  |  |  |  |  |  |  |
| ….  S068 | 1 |  |  |  |  |  |  |  |  |  |  |  |  |  |
|  | 2 |  |  |  |  |  |  |  |  |  |  |  |  |  |
|  | 3 |  |  |  |  |  |  |  |  |  |  |  |  |  |
|  | 4 |  |  |  |  |  |  |  |  |  |  |  |  |  |
|  | 1 |  |  |  |  |  |  |  |  |  |  |  |  |  |
|  | 2 |  |  |  |  |  |  |  |  |  |  |  |  |  |
|  | 3 |  |  |  |  |  |  |  |  |  |  |  |  |  |
|  | 4 |  |  |  |  |  |  |  |  |  |  |  |  |  |

**Appendix A3: Score sheet provided to professionals**

Example of AUs check in S001: Sex: Male (M) Female (F); X points to the presence of the AU.
